# Race as a determinant of clinical characteristics, treatments, and outcomes of axial spondyloarthritis in the United States

**DOI:** 10.1007/s10067-026-07974-7

**Published:** 2026-02-07

**Authors:** Catherine Bakewell, Atul Deodhar, Grace C. Wright

**Affiliations:** 1 Intermountain Healthcare, Salt Lake City, UT USA; 2 Division of Arthritis and Rheumatic Diseases, Oregon Health & Science University, Portland, OR USA; 3 Association of Women in Rheumatology, Grace C Wright MD PC, 345 E 37th Street, Suite 303C, New York, NY 10016 USA

**Keywords:** Ankylosing spondylitis, Axial spondyloarthritis, Health disparities, Health equity, Race, Racial differences

## Abstract

Axial spondyloarthritis (axSpA) is a chronic, inflammatory, immune-mediated disease characterized by inflammation of the axial skeleton, peripheral joints, and entheses. Patients with axSpA often experience a long diagnostic delay, and if left untreated, axSpA can lead to a substantial disease burden and permanent disability. In the US, axSpA is more commonly reported in White individuals than non-White individuals because of its strong association with the HLA-B27 allele, which is more common in White populations and certain Native American tribes. Underrecognition of the disease in non-White patient groups may contribute to underreporting of prevalence, diagnostic delay, undertreatment, and unnecessary disease burden in these patient populations. The goal of this review is to increase awareness and educate healthcare professionals on axSpA in non-White patients by reviewing epidemiology, diagnostic delay, genetic aspects, disease presentation, and treatment disparities among non-White patient populations in the US to promote timely recognition and treatment of axSpA in these patients.

## Introduction

Axial spondyloarthritis (axSpA) is a chronic, inflammatory, immune-mediated disease that primarily affects the sacroiliac joints and spine. The most prominent symptoms of the disease are chronic, inflammatory low back pain and prolonged morning stiffness, which improves with exercise but not with rest [1]. AxSpA is strongly associated with the class I major histocompatibility complex (MHC) allele HLA-B27, and prevalence rates of the disease are highly correlated to the frequency of HLA-B27 [2–4]. The term axSpA encompasses both radiographic axSpA (r-axSpA; also known as ankylosing spondylitis [AS]) and non-radiographic axSpA (nr-axSpA) [1, 5]. r-AxSpA is defined by definitive radiographic sacroiliitis; however, radiographic progression may occur in patients with nr-axSpA [6]. This spectrum of disease manifestations leads to underrecognition and diagnostic delay across patient populations [7], with patients experiencing a mean delay of 6.7 to 14 years globally [8–10]. This diagnostic delay is associated with worse patient outcomes, more structural damage, decreased mobility, and negative impacts on psychosocial health [11, 12]. Furthermore, delayed diagnosis may postpone the initiation of appropriate treatment, which can result in a substantial disease burden and permanent disability [11, 12]. New treatments, such as biologic disease-modifying anti-rheumatic drugs (bDMARDs) and targeted synthetic disease-modifying anti-rheumatic drugs (tsDMARDs), have demonstrated significant efficacy in improving symptoms, quality of life, and psychosocial health as well as reducing progression of structural damage and disability [1, 13].

Health disparities and inequities are evident in the US, particularly in racial minorities, driven by a combination of structural and socioeconomic factors [14]. Disparities in diagnosis, treatment, and access to care have been reported in non-White individuals with axSpA in the US, which may lead to poorer health outcomes in these populations. The prevalence of axSpA has been reported to be high in US Native American and White populations [15–18]. AxSpA is also reported to be more common in White individuals than other racial groups in the US [17], which is likely due to the high prevalence of HLA-B27 in the White population [19]. This association between White race and HLA-B27 positivity may contribute to an underrecognition by clinicians of axSpA in non-White patients and an underreporting of prevalence, diagnostic delay, undertreatment, and unnecessary disease burden in non-White patient populations. Published data on axSpA in minority racial groups in the US are limited; there is an unmet need to conduct research and educate clinicians on the prevalence and characteristics of axSpA in these underrepresented groups. The primary objective of this review is to increase awareness and to educate rheumatologists and clinicians on axSpA in non-White patients by reviewing epidemiology, diagnostic delay, genetic aspects, disease presentation, and treatment disparities among underrepresented racial groups in the US to promote timely recognition and treatment of axSpA in these groups. In this review, we use the terms White and non-White to describe overarching patient populations with axSpA in the US. When data are available, we report on specific racial and ethnic groups in the non-White category to provide a more detailed understanding of the disparities faced by various underrepresented patient populations, and when referencing a specific study, we have retained the language used by that study to define patient groups.

### Statement of literature search

Articles were identified by a series of PubMed searches between April and November 2025. Search terms included “axSpA AND race,” “axSpA AND disparities,” “axSpA AND race disparities,” “radiographic axSpA AND race,” “nonradiographic axSpA AND race,” “ankylosing spondylitis AND race,” “ankylosing spondylitis AND disparities,” “ankylosing spondylitis AND race disparities,” “axSpA AND genetics AND race,” “axSpA AND prevalence AND race,” “axSpA AND diagnostic delay AND race, “axSpA AND disease presentation AND race,” “axSpA AND disease severity AND race,” “axSpA AND Native Americans,” “ankylosing spondylitis AND Native Americans,” axSpA AND Native Americans AND prevalence,” “ankylosing spondylitis AND Native Americans AND prevalence,” “axSpA AND ethnicity AND United States,” and “ankylosing spondylitis AND ethnicity AND United States.” US-based publications that described race differences in axSpA genetics, prevalence, diagnostic delay, disease presentation, symptoms, disease severity, disease progression, comorbidities, or prescribed and used treatments were included. Articles not meeting these criteria were excluded. References cited within the included articles and articles previously known to the authors were considered based on these criteria.

### Genetic aspects

High heritability and a strong association with the HLA-B27 allele are key genetic underpinnings of axSpA [2–4]. Although HLA-B27 is significantly associated with axSpA, it does not fully account for the genetic basis of the disease. Approximately 25% of patients with axSpA are HLA-B27 negative [3, 20, 21], and only 1% to 5% of individuals who are HLA-B27 positive go on to develop the disease [22, 23]. Over 100 MHC and non-MHC variants associated with axSpA have been identified through genome-wide association studies, including *ERAP1* variants and multiple variants involved with the IL-23 pathway genes [2, 24–27]. Moreover, HLA-B27 only contributes to approximately 20% of axSpA heritability [24], and the additional axSpA-associated MHC and non-MHC variants are thought to account for less than 10% of its heritability [3, 24]; thus, much of the genetic underpinnings of axSpA pathogenesis is still unknown.

Historically, research efforts on the genetic basis of axSpA have focused on the HLA-B27 allele, which varies in prevalence across racial/ethnic groups (Table 1) [19, 28]. In a US study, using data from the 2009 National Health and Nutrition Examination Survey (NHANES), the HLA-B27 allele was more prevalent in White Americans (7.5%) than non-White Americans (3.5%) [19]. Additionally, the prevalence rate in Mexican Americans (4.6%) was significantly lower than in non-Hispanic White Americans (7.5%), while prevalence rates of HLA-B27 in other non-White groups could not reliably be estimated due to small sample sizes [19]. The prevalence of HLA-B27 is also thought to be high in Alaska Natives and Native Americans, with reports of an HLA-B27 prevalence of 25% and 40% in the Yupik and Inupiat Eskimos, respectively [29], 9% in Hopi Americans, and 36% in Navajo Americans [30]. A study of Native American patients in Arizona found that 100% of patients with AS were positive for HLA-B27 [16]. However, these studies were conducted in the 1980s and 1990 s, and current prevalence estimates of HLA-B27 in Alaska Natives and Native Americans with axSpA are unknown.

**Table 1:** Differences in axSpA clinical characteristics, presentation, and treatment across racial groups in the US

Characteristics	Alaska Native	Native American	Asian	Black/African American	Hispanic/Latino	Native Hawaiian or Other Pacific Islander	White	References
**HLA-B27 prevalence, %**								
In population	25.0–40.0	9.0–36.0	-	-	4.6^a^	-	7.5	[16, 19, 29, 30]
In patients with axSpA	-	100.0	-	42.0–62.5	86.7	-	64.0- 85.3	[16, 28, 31, 32]
**Prevalence rates of axSpA, %**								
Population prevalence rate	0.1–0.5	2.0	-	-	-	-	1.5	[15, 16, 18]
Diagnostic prevalence rate in patients with commercial insurance	-	-	0.07	0.08	0.08	-	0.12	[17]
Diagnostic prevalence rate in patients with Medicaid	-	-	-	0.04	0.05	-	0.09	[17]
Diagnostic prevalence rate in patients with Medicare	-	-	0.11	0.12	0.16	-	0.16	[17]
**Diagnostic delay, mean, years**	-	-	-	7.9	-	-	6.6	[28]
**Disease presentation**								
Rate of psoriasis in patients with axSpA, %	-	-	-	1.3–6.5	-	-	10.4–11.0	[28, 31]
Age at symptom onset, mean, years	-	-	-	26.8–29.8	23.8	-	24.0–27.3	[28, 32]
CRP level, median, mg/dL	-	-	-	1.2	0.9	-	0.4	[32]
ESR, median, mm/h	-	-	-	27.0	10.0	-	17.0	[32]
BASDAI (0–10) score, median	-	-	-	5.9	4.5	-	3.5	[32]
BASFI (0–100) score, median	-	-	-	62.5	38.1	-	27.8	[32]
BASRI (1.5–16) score, median	-	-	-	10.3	7.5	-	7.0	[32]
mSASSS (0–64) score, median	-	-	-	38.2	8.1	-	6.4	[32]
Rate of hip involvement, %	-	-	-	48.0	-	-	34	[28]
**Treatment rates, %**								
TNFi	-	-	-	26.0–37.0	36.8	-	40.0–42.0	[31, 32]
bDMARD/tsDMARDS in patients with commercial insurance	-	-	44.5	41.8	38.0	-	43.8	[17]
bDMARD/tsDMARDS in patients with Medicaid	-	-	-	13.4	23.5	-	20.6	[17]
bDMARD/tsDMARDS in patients with Medicare	-	-	7.7	13.9	12.3	-	10.5	[17]
AxSpA drug trials	2.0 Alaska Native/Native American		15.0	1.0	14.0	0.02	82.0	[64]

^a^ In Mexican Americans

*AxSpA* axial spondyloarthritis, *BASDAI* Bath Ankylosing Spondylitis Disease Activity Index, *BASFI* Bath Ankylosing Spondylitis Functional Index, *BASRI* Bath Ankylosing Spondylitis Radiographic Index, *bDMARD* biologic disease-modifying anti-rheumatic drug, *CRP* C-reactive protein, *ESR* erythrocyte sedimentation rate, *mSASSS*, modified Stoke Ankylosing Spondylitis Spine Score; *TNFi* tumor necrosis factor inhibitor, *tsDMARD* targeted synthetic disease-modifying anti-rheumatic drug

White patients with axSpA are also more likely to be positive for HLA-B27 than Black patients with axSpA. Significantly higher HLA-B27 positivity was observed among White Americans with AS vs Black Americans with AS (64% vs 42%, *P* = 0.0018) in an analysis of data from 101 patients [31] and among White Americans with axSpA vs Black Americans with axSpA (77% vs 59%, *P* = 0.010) in a separate study of electronic health records (EHRs) from 244 patients [28]. Additionally, an analysis of 925 patients with AS enrolled in the longitudinal Prospective Study of Outcomes in AS showed a significantly higher frequency of HLA-B27 in White (85.3%) and Latino (86.7%) patients than in Black patients (62.5%) (*P* < 0.0001) [32].

Previous studies have demonstrated a positive association between HLA-B27 and disease activity in patients with AS [33–35]; however, in separate studies, a negative association between HLA-B27 and disease activity was demonstrated in patients with axSpA [36, 37]. This paradoxical difference was hypothesized to result from axSpA encompassing nr-axSpA and r-axSpA—a broader patient population—compared with AS (r-axSpA) [38]. This also may be related to a cognitive bias towards diagnosing patients with back pain who are HLA-B27 positive as having axSpA [36]. In addition, Black patients with axSpA, who are more likely to be negative for HLA-B27, have higher disease activity and more severe disease than non-Black patients, highlighting a potential consequence of the reliance on HLA-B27 in the diagnosis of axSpA [28, 31, 32].

Although genetic differences between racial subgroups with axSpA are not well understood, a few studies have identified alleles that may be associated with the disease in certain racial subgroups. A previous study examined the association of HLA class I and II alleles with AS in three ethnic groups (African American, Han Chinese, and White). In all three ethnic groups, HLA-B*27 was the major genetic association with AS [26]. HLA-A*29, B*38, B*49, B*52, DRB1*11, and DPB1*03:01 were positively associated with AS in White patients who were HLA-B27 negative; however, associations between HLA alleles and AS were not examined in African American or Han Chinese patients who were HLA-B27 negative. Positive associations were also observed with HLA-B*40:01 in African American, Han Chinese, and White patients as well as HLA-B*14 in White patients [26]. In a 1978 study, a high frequency of HLA-B7 was observed in American Black patients with AS who were HLA-B27 negative [39], but an association between HLA-B7 and AS in African American patients was not observed in a 2019 study [26]. Additionally, MHC class I chain-related gene A (*MICA*)*007:01 and MICA*019 were previously identified as susceptibility alleles for AS in Han Chinese patients, and MICA*007:01 was identified in European White patients [40]. Overall, there is a lack of genetic data for non-White patients with axSpA, likely contributing to a disparity in diagnosis.

### Epidemiology and diagnostic delay

In the US, prevalence of axSpA varies across racial groups, and sex-based differences in the prevalence rate of AS are also well documented [41, 42]. Sex differences in axSpA have also been comprehensively reviewed in our previous study [38]. The overall population prevalence of axSpA (prevalence of diagnosed and undiagnosed axSpA) is estimated to range from 0.9% to 1.4% in the general US adult population, based on data from the 2009–2010 NHANES [18]. The NHANES reported a population prevalence of 1.5% in White individuals, but prevalence estimates in other racial groups could not conclusively be determined due to low sample sizes (Table 1) [18]. A retrospective study of EHRs reported a diagnostic prevalence (prevalence of diagnosed axSpA) of 0.2% in the US adult population; however, diagnostic prevalence by race was not examined [43]. In an analysis of 10,990 patients with AS using EHRs from the Explorys platform, a higher percentage of patients were White (84.3%) vs non-White [31], and in a study of 244 patients with axSpA, the majority of patients were White (59%) [28]. The diagnostic prevalence of AS by insurance type, race, and sex was estimated in a study of US claims data that found an overall higher prevalence in White patients enrolled in commercial insurance (0.12%) or Medicaid (0.09%) compared with non-White patients enrolled in commercial insurance (Asian, 0.07%; Black, 0.08%; Hispanic, 0.08%) or Medicaid (Black, 0.04%; Hispanic, 0.05%) [17]. Among patients enrolled in Medicare, the highest prevalence of AS was observed in White and Hispanic patients (0.16% for both) compared with other groups (Asian, 0.11%; Black, 0.12%) [17]. When the data were examined by sex, males with Medicare or Medicaid had higher prevalence rates of AS (0.20% and 0.09%, respectively) than females (0.13% and 0.06%), with similar trends across racial groups. In patients with commercial insurance, similar prevalence rates were observed between males and females (0.10%); however, Asian males had higher rates than Asian females, while Hispanic males had lower rates than Hispanic females. Overall, White males enrolled in Medicare had the highest prevalence rate (0.21%) and Black females enrolled in Medicaid had the lowest (0.04%) [17].

High prevalence rates of spondyloarthritis have been reported in the indigenous populations of North America [15, 16]. Prevalence rates of spondyloarthritis range from 1.0% to 2.7% in certain Alaska Native communities, and the prevalence of AS in Alaska Natives is reported to range from 0.1% to 0.5% [15]. High prevalence of radiographic sacroiliitis, a distinguishing feature of r-axSpA, has been reported in various Native American populations, with 11% of Navajo, 4% of Hopi, 11% of Pima, and 2.5% of Blackfeet patients (males only) reported to have grade ≥ 2 sacroiliitis [16, 30]. A study following approximately 700 Native American patients in Arizona found that 110 (16%) had spondyloarthritis, with 15 (2%) reported to have AS [16]. The reported high prevalence of sacroiliitis and spondyloarthritis in Alaska Native and Native American patients is likely due to the high prevalence of HLA-B27 in these populations [16, 30]. Most prevalence studies in Native American populations were conducted approximately 30 to 50 years ago and only in certain groups; current prevalence rates of axSpA in Native American populations are unknown. Overall, existing evidence indicates that axSpA is more prevalent among White Americans and Native Americans, but limited data in underrepresented racial groups underscore a substantial knowledge gap and the need for more inclusive studies.

**Fig. 1:**
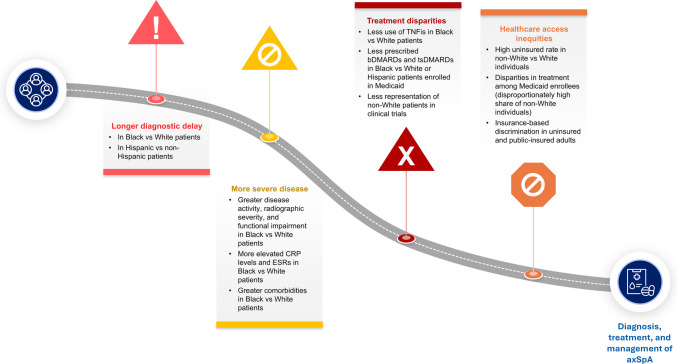
Patient journey to axSpA diagnosis and treatment for non-White patients in the US. *AxSpA* axial spondyloarthritis, *bDMARD* biologic disease-modifying anti-rheumatic drug, *CRP* C-reactive protein, *ESR* erythrocyte sedimentation rate, *TNFi* tumor necrosis factor inhibitor, *tsDMARD* targeted synthetic disease-modifying anti-rheumatic drug

Previous studies have shown longer diagnostic delays in women vs men [38, 44] and in Black vs White patients and Hispanic/Latinx vs non-Hispanic/Latinx patients [28, 45], potentially leading to the underdiagnosis of the disease in women and non-White patients (Table 1 and Fig. 1). A study of 244 patients with axSpA showed that Black Americans had a numerically longer delay in diagnosis vs White Americans (7.93 vs 6.64 years) [28]. Factors contributing to this longer delayed diagnosis included a lack of access to advanced imaging such as magnetic resonance imaging and a reliance on HLA-B27 for diagnosis; most patients in the study were diagnosed with r-axSpA based on plain radiographs, and a significantly lower percentage of Black Americans (59%) were HLA-B27 positive compared with White Americans (77%) [28]. In addition, multiple studies have demonstrated a significant association between HLA-B27 negativity and delayed diagnosis in patients with axSpA [35, 46, 47]. Diagnostic delays observed in non-White populations and women may lead to systematic underestimation of axSpA prevalence in these groups. Recognizing and addressing these diagnostic disparities is critical to improving timely identification and treatment of axSpA in women and non-White individuals.

### Variations in disease presentation

Patients with axSpA from different racial/ethnic backgrounds may exhibit varying disease presentations, including differences in symptoms, disease severity, and age of onset (Table 1 and Fig. 1). White patients with axSpA have significantly higher rates of psoriasis vs Black/African American patients with axSpA (10.4%−11.0% vs 1.3%−6.5%) [28, 31]. Age of axSpA symptom onset is later in Black/African American patients (mean, 26.8–29.8 years) than in Latino (mean, 23.8 years) and White patients (mean, 24.0–27.3 years) [28, 32]. Greater comorbidities have been reported in African American patients with AS compared with White patients, including a higher frequency of hypertension (29.3% vs 21.7%), heart disease (24.0% vs 21.7%), diabetes (27.2% vs 17.3%), and depression (35.8% vs 31.9%) [31]. A limitation of this study is the absence of matched controls without AS, which prevents determination of whether the comorbidity rate observed in African Americans with AS is increased compared to those without AS. Nonetheless, hypertension, heart disease, and diabetes are more prevalent in African American populations than in White populations [48, 49], making it likely that AS adds to an already elevated chronic disease burden in African Americans. Additionally, two studies reported higher rates of anterior uveitis in Black/African American patients with axSpA than in White patients, with one study reaching statistical significance [28, 31]; however, a third study reported similar rates of anterior uveitis in Black, Latino, and White patients with AS [32]. Anterior uveitis is strongly associated with HLA-B27 positivity [50]; despite a higher prevalence of HLA-B27 in White individuals, studies have not demonstrated a higher prevalence of anterior uveitis in White patients with axSpA compared with non-White patients [28, 31, 32]. Data on disease severity and presentation in Native Americans are lacking, despite a reportedly high prevalence of axSpA in certain Native American and Alaska Native tribes [15, 16, 30].

Black/African American patients with axSpA often have higher levels of inflammatory markers, compared with other racial groups [28, 31, 32]. In an analysis of AS presentation and severity in three racial/ethnic groups, baseline C-reactive protein (CRP) levels were higher in Black patients with AS (median, 1.2 mg/dL) vs Latino (median, 0.9 mg/dL) and White (median, 0.4 mg/dL) patients with AS (*P* < 0.0001), and baseline erythrocyte sedimentation rate (ESR) was also higher in Black patients (median, 27 mm/h) vs Latino (median, 17 mm/h) and White (median, 10 mm/h) patients (*P* < 0.001) [32]. In a 2020 retrospective analysis of EHRs from 10,990 patients with AS, higher proportions of African American patients had elevated CRP levels and ESRs vs White patients (elevated CRP, 67.7% vs 54.4%; elevated ESR, 61.5% vs 47.6%; *P* < 0.0001 for both) [31]. Furthermore, a 2025 study showed significantly higher rates of elevated CRP levels in African American patients with axSpA compared with White patients (66% and 46%, respectively) [28].

Greater disease activity, severe disease manifestations, and functional impairment have been reported in Black/African American patients with axSpA compared with White and Latino patients. Higher disease activity, as measured by the Bath Ankylosing Spondylitis Disease Activity Index (BASDAI), was previously observed in Black patients with AS (median, 5.9) compared with Latino (median, 4.5) and White (median, 3.5) patients with AS (*P* < 0.0001), as well as significantly greater radiographic severity [32]. Additionally, greater functional impairment as measured by the Bath Ankylosing Spondylitis Functional Index (BASFI) was observed in Black patients with AS (median, 62.5) compared with Latino (median, 38.1) and White (median, 27.8) patients with AS (*P* < 0.0001) [32]. An analysis of 244 patients with axSpA found that Black Americans had a significantly higher rate of hip involvement than White Americans (48% and 34%, respectively; *P* = 0.04) and higher odds of having advanced grades of sacroiliitis on x-rays (odds ratio, 2.32 [95% confidence interval, 1.23–4.44]) [28]. Overall, elevated CRP levels and ESRs along with worse disease activity, radiographic severity, and functional impairment in Black/African American patients with axSpA compared with White and Latino patients suggest more severe disease in Black patients. The expectation that axSpA primarily affects White patients may lead to more frequent and earlier diagnoses in this group, including diagnosis of milder cases, while in non-White patients the disease may go unrecognized until it becomes more severe [32]. This may partially explain the difference in disease severity seen between Black and White patients.

### Social determinants of health

The disparities observed between White and non-White patients with axSpA may partially be explained by social determinants of health. Factors such as income, education, employment, housing, and healthcare access play a role in health and quality-of-life outcomes and disproportionately affect people of color and indigenous individuals [14]. A 2025 study in the US showed that patients with a documented social need had a 21% increase (approximately 7-month increase) in time from back pain to diagnosis of r-axSpA [45]. This study also demonstrated a longer diagnostic delay in non-White patients (Black and Hispanic/Latinx) vs White patients [45]. The authors of this study called for the incorporation of a tailored curriculum in medical schools and residency training programs to help healthcare professionals in training develop a standardized approach to recognizing social determinants of health in patients to reduce disparities in axSpA care [45].

Non-White patients with axSpA may experience barriers to healthcare access and care due to disparities in insurance coverage. Healthcare costs for patients with AS are high ($24,978 per patient per year vs $2139 per patient per year in matched controls) [51], creating substantial financial barriers to care. In the US, the groups with the highest uninsured rate in 2022 were Native American/Alaska Native (18.8%) and Hispanic/Latino (17.7%), and the groups with the lowest uninsured rates were White (5.7%) and Asian (5.8%) (Fig. 1) [52]. The uninsured rate of Black individuals (9.6%) was also higher than White and Asian individuals in 2022 [52]. The low uninsured rate in Native Americans/Alaska Natives along with making up only 0.7% of the US population [53] may explain why data on axSpA in Native Americans/Alaska Natives are limited as fewer Native Americans may interact with the US healthcare system compared with individuals of other racial groups. Limited insurance coverage among some non-White populations (including Native American/Alaska Native, Hispanic/Latino, and Black communities) may restrict access to sacroiliac joint imaging, leading to the underdiagnosis and underreporting of axSpA and the skewing of prevalence estimates.

Black, Hispanic, and Native American/Alaska Native individuals make up 21%, 28%, and 1.3% of the Medicaid population, respectively, despite only representing 12%, 19%, and 0.7% of the US population [53]. White Americans represent 60% of the US population but only make up 43% of the Medicaid population [53]. A previous study showed greater insurance-based discrimination in uninsured and public-insured adults compared with those who had private insurance, and patients who experienced insurance-based discrimination vs those who did not were more likely to lack a consistent source of care and forgo needed care because of cost [54]. In an analysis of racial differences in AS treatment by insurance coverage, the proportions of patients with AS prescribed bDMARDs and tsDMARDs were higher in commercial insurance enrollees (43%) than Medicare (11%) or Medicaid (20%) enrollees [17]. Visits to rheumatologists were also lower in Medicaid enrollees with AS (10%) compared with those patients with commercial insurance (67%) or Medicare (42%) [17].

### Treatment disparities

Several studies have reported differences in the treatment and management of axSpA and back pain between White and non-White patient groups (Table 1 and Fig. 1). An analysis of EHR data demonstrated a trend toward less use of tumor necrosis factor inhibitors in African American patients with AS compared with White patients (37% and 40%, respectively; *P* < 0.08) [31]. In addition, a trend in less use of tumor necrosis factor inhibitors among Black patients compared with White and Latino patients was shown in a study of patients enrolled in the longitudinal Prospective Study of Outcomes in AS [32]. Furthermore, in a study of 18,318 US veterans with inflammatory arthritis (including ankylosing spondylitis, rheumatoid arthritis, and psoriatic arthritis), Black race and Hispanic ethnicity were associated with not receiving biologic or non-biologic DMARD treatment (hazard ratio, 95% CI: 1.13, 1.08–1.19 and 1.14, 1.07–1.22, respectively) [55]. In an analysis of differences in AS treatment by race, sex, and insurance coverage, the proportions of patients who were prescribed bDMARDs and tsDMARDs were similar across racial/ethnic groups for those enrolled in commercial insurance or Medicare but lower for Black patients enrolled in Medicaid (13%) vs White (21%) and Hispanic (24%) patients enrolled in Medicaid [17]. Among patients with commercial insurance, the proportion of females receiving bDMARDs and tsDMARDs was lower than males (38% and 47%, respectively). The percentage of women vs men receiving bDMARDs and tsDMARDs was similar for patients enrolled in Medicare (11.3% vs 10.7%) and slightly lower for those enrolled in Medicaid (18% vs 21%). Similar trends were observed across racial groups [17]. The lower use of biologics among Black/African American patients with axSpA may stem from several factors such as reduced prescribing by clinicians because of implicit biases, existing comorbidities, patient medical mistrust, and patient-perceived discrimination [56, 57].

Differences in the treatment of back pain, a primary symptom of axSpA, have also been reported across racial groups. Non-White Americans, including Asian, Black, Hispanic, or Pacific Islanders, are less likely to be prescribed opioid analgesics and more likely to be prescribed nonsteroidal anti-inflammatory drugs for back pain than White Americans [58, 59]. This has been partly attributed to a lower likelihood of non-White patients requesting opioid medications and potential differences in physician trust [58, 59]. Black patients are also less likely to receive early spine imaging for back pain than White patients, and both Black and Hispanic patients have less spine-related healthcare utilization for back pain than White and non-Hispanic patients, respectively [60]. Nonetheless, lumbar spine imaging is not recommended in patients with acute or chronic low back pain when no complications or “red flags” are present [61]. Additionally, not receiving opioids or routine spine imaging is consistent with the 2019 American College of Rheumatology/Spondylitis Association of America/Spondyloarthritis Research and Treatment Network guidelines, which recommend against opioid analgesics and against scheduled interval spine imaging (particularly lumbar imaging) for the treatment and management of axSpA [62]. When axSpA is suspected, sacroiliac joint imaging is a key diagnostic method and a central tool of patient evaluation [62, 63]. However, these previous studies of back pain treatment by race did not document diagnostic evaluations for specific diseases [58–60]. The reasons for differences in healthcare utilization in non-White patients with back pain are unclear but may reflect broader health inequities [14, 60].

Racial representation in clinical trials of axSpA is unbalanced, with low representation of Black and Native Hawaiian/Pacific Islander participants in axSpA drug trials compared with other groups. An analysis of diversity in axSpA drug trials reported that of 10,037 participants with race data, only 1% were Black, and 0.02% were Native Hawaiian/Pacific Islander, while 82% were White, 15% were Asian, and 2% were Native American/Alaska Native [64]. Moreover, Black representation has continually stayed low (at 1%) from 2011 to 2020, while representation of Asian and Native American/Alaska Native patients has increased (4% to 19% and 1% to 3%, respectively, from 2011–2016 to 2016–2020) [64]. In studies with ethnicity data reported, 14% of participants were Hispanic/Latino in 2016–2020, which increased from 1% in 2011–2016 [64]. Future axSpA clinical trials should prioritize enrolling underrepresented patient populations.

## Conclusion

Notable differences in axSpA exist between White and non-White patients in the US, including in prevalence, diagnostic delay, genetic factors, disease presentation and severity, and treatment. While disparities between White and Black Americans are well characterized, far less is known about other underrepresented groups, including Alaska Natives, Native Americans, Asian Americans, and Native Hawaiian/Other Pacific Islanders. Inclusive, large-scale studies are needed to better understand the factors contributing to the disease, identify disparities, improve health equity, and guide more effective care for all patient populations with axSpA in the US.
